# COVID-19 pandemic and access to mental healthcare: A qualitative study of the experiences of mental healthcare providers and caregivers in Ghana

**DOI:** 10.1371/journal.pmen.0000386

**Published:** 2025-07-29

**Authors:** Leonard Baatiema, Olutobi A. Sanuade, Sheba M. P. Kunfah, Kwaku Darko Owusu-Ansah, Luke N. Allen, Benedict Weobong, Seye Abimbola, Ama de-Graft Aikins, Kwadwo A. Koram, Margaret E. Kruk

**Affiliations:** 1 Department of Health Policy, Planning and Management, School of Public Health, University of Ghana, Legon, Ghana; 2 Department of Global Health and Population, Harvard T.H. Chan School of Public Health, Boston, Massachusetts, United States of America; 3 Department of Population Health Sciences, Spencer Fox Eccles School of Medicine, University of Utah, Salt Lake City, Utah, United States of America; 4 Global Primary Care, Nuffield Department of Primary Care Health Sciences, University of Oxford, London, United Kingdom; 5 School of Global Health, Faculty of Health, York University, Toronto, Ontario, Canada; 6 School of Public Health, Faculty of Medicine and Health, University of Sydney, Australia; 7 Regional Institute for Population Studies, University of Ghana, Legon, Ghana; 8 Department of Methodology, London School of Economics and Political Science, London, United Kingdom; 9 Noguchi Memorial Institute for Medical Research, University of Ghana, Legon, Ghana; PLOS: Public Library of Science, UNITED KINGDOM OF GREAT BRITAIN AND NORTHERN IRELAND

## Abstract

The COVID-19 pandemic created enormous additional demand for already weak mental health systems in many countries that were severely impacted, as evidence showed that new mental health disorders were triggered while those with pre-existing mental health conditions worsened. Yet, we know little about the extent to which the pandemic impacted the provision of care by frontline mental health care providers and access to mental health care among caregivers. This study describes the experiences of frontline mental health care providers and caregivers during the COVID-19 pandemic in Ghana. A qualitative study employing a phenomenological approach and semi-structured interview methods was undertaken among caregivers of people living with any kind of mental illness and mental health care providers. We used a purposive sampling technique to select study participants who were available and consented to share their experiences. An interview guide facilitated the data collection process. We used an inductive approach through open coding to generate codes. We then grouped codes into emergent categories and further into themes of experiences of accessing mental health services by users and provision of mental health services by providers. Our analysis identified six themes regarding frontline mental health care providers’ experiences at the primary health care level during the COVID-19 pandemic: a) interrupted home visits b) lack of government support c) lack of medication d) low clinic attendance e) disruptions in service delivery f) extended medication supply and g) patient aggression and attacks. Four themes emerged that characterize the experiences of caregivers of people living with mental health conditions, namely a) denial and withdrawal of facility-based care b) medication refill challenges c) disruption in outreach and follow-up calls and d) isolation and limited community support. The study highlights the challenges experienced by frontline mental health care providers and caregivers of people living with mental health conditions. It brought to light specific gaps that require urgent attention for people living with mental health conditions, their caregivers, and frontline workers. Institutional additional support systems to ensure fewer disruptions and address access-based gaps through outreach and community support would help improve their experiences in future pandemics.

## Background

Access to mental health services is a major global health challenge that has existed for decades [1]. Sub-Saharan Africa bears the heaviest burden of this challenge, resulting from factors including but not limited to poorly funded public health systems, stigmatization and discrimination of persons living with mental illnesses and their relatives, poor and inadequate infrastructure for mental health and insufficiency of mental health professionals. These have led to major gaps in managing and treating mental illnesses in SSA countries [2,3]. The unprecedented COVID-19 pandemic created an enormous additional demand for the already weak mental health systems in many countries [4]. For example, in the wake of the pandemic, new mental health disorders were triggered while those with pre-existing mental health conditions worsened [5]. A global scoping review highlighted that people with a known history of mental health conditions had aggravated symptoms, were more likely to have a relapse of their conditions and were at risk of worsening psychological distress [6]. The introduction of safety measures of the pandemic, including lockdown restrictions, travel restrictions and even restrictions on hospital attendance, disrupted routine social and economic activities [7]. This led to increased anxiety, depression, feelings of hopelessness, fear, sleeplessness, and panic attacks, among other mental health conditions [6,8,9].

Although there was an increased demand for mental health services during this period, the provision of important mental health services (e.g., for anxiety, schizophrenia and depression) declined greatly as reported by 93% of countries. Mental healthcare providers who provide mental health preventive and promotive services and community-based mental health services in many countries were also negatively impacted. Undoubtedly, the pandemic impacted people living with mental health conditions, including their caregivers (CG), who provided care and support for the clients at home. For example, Yusif et al reported that the workload and stress levels of caregivers of mental health patients increased during this period as caregivers had to take up the additional role of providing rehabilitation services for their patients due to the disruptions and closure of major mental health clinics across health facilities [10]. There was also an extra burden of protecting themselves and their patients from COVID-19 by implementing infection prevention practices [10]. This worsened the burden of caregivers to continuously care for the patient with no periods of rest, resulting in emotional and physical stress [11]. Caregivers experienced inadequate access to social support, and some lost their jobs or struggled to provide for basic family needs [12]. Social isolation during COVID-19 resulted in long periods of loneliness [13], leading to anxiety, depression and sleeplessness among caregivers [11].

In Ghana, a recent report has observed that about 2.6 million people living in Ghana (10.7% of the country’s population) have a mental health condition (e.g., schizophrenia, bipolar disorder, or major depressive disorder) and yet there is a substantial unmet need; only 2% of primary are attendees have their Mental Neurologcal and Substance condition detected [14], and about 2% receive treatment for their conditions [15] Despite this high burden of mental health illness in the population, access to mental healthcare has remained a fundamental health system challenge [16,17]. Access is limited, with only a few psychiatric hospitals in the country and all three major psychiatric hospitals are located in the southern part of the country, depriving and restricting access to mental health care by the rest of the population [18,19]. Community mental health systems have been developed with the training of community mental health nurses to improve access to care through supporting outreach visits and treatment services in communities [20,21]. However, access remains a challenge due to the limited workforce [17,22,23].

The COVID-19 pandemic has taken a significant toll on health systems and primary caregivers of people living with mental health conditions in Ghana. However, studies to date have focused predominantly on the impact of the pandemic on the overall psychosocial well-being of the population [24–27] and the impact of the pandemic on the psychological impact and well-being of health care providers [25,28–32]. However, there is a major knowledge gap on how the pandemic impacted the delivery of mental health services and the extent to which access to these services among people living with mental health conditions and their primary caregivers was impacted by the pandemic. This study sought to describe the experiences of healthcare workers (HCWs) in providing mental healthcare services as well as CG experiences in accessing mental health (MH) services during the COVID-19 pandemic. We aimed to provide a specific perspective on the challenges faced by mental healthcare workers and caregivers in accessing mental health services during the pandemic.

## Methods

### Study design

**Fig 1:**
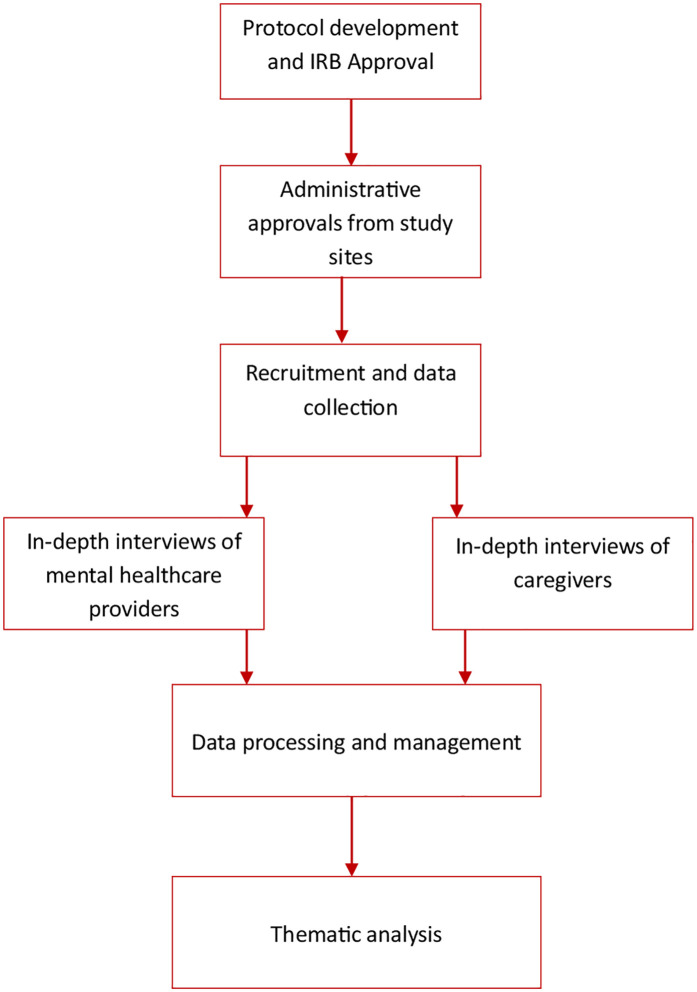
Flow chart for participants’ recruitment, data collection and analysis.

We used qualitative research employing a phenomenological approach and semi-structured interview methods. This study was part of a larger project that sought to understand how the COVID-19 pandemic impacted access to non-communicable disease care in Ghana. The study was conducted in the outpatient departments of three healthcare facilities and via telephone interviews of caregivers in the Northern, Ashanti and Greater Accra regions of Ghana. An overview of the study flow chart is presented in Fig 1.

### Study setting and population

The study was conducted in three of Ghana’s sixteen administrative regions: the Northern, Ashanti, and Greater Accra regions. These regions were purposively selected based on their locations in the northern, middle and southern ecological zones of Ghana. Tamale Teaching Hospital, Komfo Anokye Teaching Hospital and Achimota Hospital were facilities in which the study was conducted in the Northern, Ashanti and Greater Accra regions, respectively. In this study, we interviewed caregivers of people living with any kind of mental illness and mental health care providers. The focus was to elicit primary caregiver perspectives on access to care for their family member with a mental health condition. As a result, we focused on caregivers of patients with severe mental illness. We did not interview patients, as healthcare providers recommended that interviews may cause distress.

### Sampling and recruitment approaches

We used a purposive sampling technique to select caregivers and mental healthcare providers who were available and consented to share their experiences on providing mental health services during the COVID-19 pandemic. To ensure diversity, we recruited both male and female caregivers from different age categories. Mental healthcare providers of different sexes and years of professional experience were also recruited to optimize diversity.

The caregivers were identified in two ways. First, we recruited caregivers during outpatient department visits before or after consultation. We identified and recruited the caregivers through the mental health outpatient department heads and managers, or the mental health care providers who facilitated the recruitment process. Before this, the caregivers and mental health unit heads or in-charges were informed about the study. Secondly, the staff of the outpatient units made contact with the caregivers via telephone calls to inform them about the study and the option to be interviewed either via telephone or in person. Those who agreed to be contacted had their contacts shared with the research team and the necessary follow-up was carried out. As regards the mental health care providers, recruitment and interviews were done at the various OPDs for those who expressed interest in participating in the study.

### Data collection and sample size

The interview guide was developed based on relevant literature and experience, and knowledge of the research team on the subject matter. Three research assistants conducted qualitative interviews with caregivers and mental healthcare providers from 10^th^ March to 8^th^ June 2022. Although the data collection faced several postponements as requested by the caregivers, we provided much flexibility to ensure the interviews were conducted at the convenience of the participants due to the extra workload and caregiving burden occasioned by the COVID-19 pandemicData collection was done at the study sites and remotely via telephone calls based on the preferences of the interviewees. For mental health care providers, questions were asked about their experiences, patient and health care system challenges they faced in providing health care services during the COVID-19 period. For the caregivers, questions were also asked about their experiences and the challenges faced in receiving care for their patients and providing support to their patients during the COVID-19 pandemic. All interviews were conducted in English for the health workers and some caregivers. However, the caregivers preferred to be interviewed in the local languages, namely Dagbani and Twi. On average, the interviews lasted about 35 minutes (30–40 minutes). Study participants were compensated with additional packs of face masks following the interviews. We continued recruiting participants until we reached thematic saturation. Recruitment and data analysis were performed concurrently. Data collection lasted two months.

### Data analysis

All interviews were transcribed verbatim, and thematic analysis was utilized to analyze the transcripts (Braun & Clarke, 2006). We used an inductive approach through open coding to generate codes. Using the constant comparison approach to identify similar codes, we looked for similarities and differences and emerging patterns of the codes. We then grouped codes into emergent categories, and further into themes of experiences of accessing mental health services by the users and provision of mental health services by the health care providers. Using Dedoose software (v2.0, Manhattan Beach, CA, USA), we generated initial codes, developed and reviewed the themes. We then organized the themes based on their experiences and associated challenges.

### Rigour and trustworthiness

We employed several strategies to improve the rigour and quality of the results. First, the interview schedule was pre-tested among similar participants in the study sites. However, the results were not included in this analysis, although the experiences from the exercise helped refine the schedule for the main study. We also followed the consolidated criteria for reporting qualitative research (COREQ) throughout the study design and data analysis [33]. Further, three researchers led the data analysis process to develop and refine the coding frame with later review and inputs from the other researchers to ensure rigour. Reflective and detailed notes were taken during the data collection/analysis and this guided the final interpretation of results.

### Ethics approval and consent to participate

Ethics approval was obtained from the Ghana Health Service Ethics Committee (Protocol ID NO: GHS-ERC 018/09/21). Research support letters were obtained from the host institution for this study (WACCBIP, University of Ghana) and administrative approvals from study sites were also granted before the data collection. Participants were informed about the study and subsequently consented before all interviews. All information was anonymized and deidentified during data analysis and reporting to ensure confidentiality and anonymity.

## Results

We interviewed six caregivers whose ages ranged from 21 to 58 years and ten mental health providers whose ages ranged from 28 to 36 years. The six caregivers consisted of five females and a male who were closely related to the patients. The ten mental health providers were made up of six males and four females; five of them were from the Achimota hospital (Greater-Accra region), four from the Tamale Teaching Hospital (Northern region) and one from Komfo Anokye Teaching Hospital Ashanti region). The Caregivers of the patients were either a spouse, fiancée or a first or second degree relative to the patient. Table 1 displays the characteristics of the study participants.

**Table 1:** Participants’ demographic characteristics.

Mental health care providers	Age	Sex	Qualification	Facility	Years of professional practice
MH Provider__ 1	32	F	Dip, Mental Health Nursing	Health Facility A in Ashanti Region	5
MH Provider __2	35	M	Dip. Mental Health Nursing	Achimota Hospital, Accra	5
MH Provider __3	28	M	BSc. Mental Health Nursing	Achimota Hospital, Accra	5
MH Provider __4	31	F	Dip. Mental Health Nursing BSc. Nursing	Achimota Hospital, Accra	7
MH Provider __5	36	M	Dip. Mental Health Nursing BSc. Nursing, MPhil. Nursing.	Achimota Hospital, Accra	10
MH Provider __6	29	M	Dip. Mental Health Nursing	Health Facility A in Northern Region	5
MH Provider __7	30	M	BSc. Mental Health Nursing	Health Facility A in Northern Region	4
MH Provider __8	33	F	Dip. Mental Health Nursing	Health Facility A in Northern Region	5
MH Provider __9	35	F	Mental Health Physician (Psychiatrist)	Health Facility A in Northern Region	2
MH Provider _10	34	M	BSc. Mental Health Nursing	Health Facility A in Ashanti Region	3
**Caregivers**			**Relationship to patient**		**Chronicity of MH Condition (years)**
Care Giver__1	58	F	Mother of patient		4
Care Giver__2	31	F	Niece of patient		5
Care Giver__3	21	F	Niece of patient		3
Care Giver__4	47	M	Father of patient		4
Care Giver__5	45	F	Wife of patient		6
Care Giver__6	32	F	Fiancee		5

### Summary of findings

**Fig 2:**
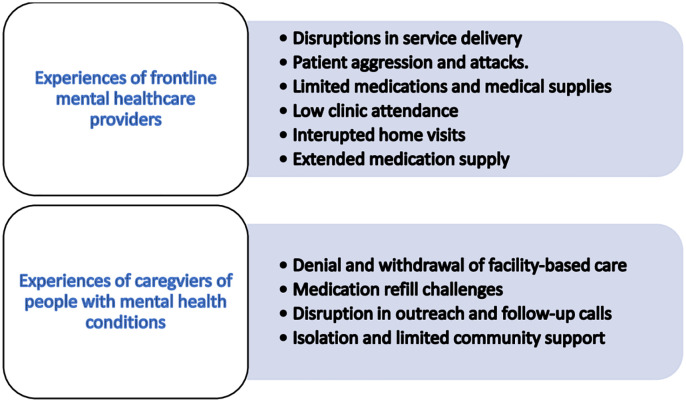
An overview of the themes from the qualitative analysis.

Here we present findings from an analysis of in-depth interviews with frontline mental healthcare providers and the caregivers of people living with mental health conditions. For the frontline mental healthcare providers, we identified six main themes whilst four main themes were found documenting the experiences of providing mental health care and access to these services respectively, during the COVID-19 pandemic. Fig 2 presents an overview of the themes and sub-themes.

### Frontline mental healthcare providers

Our analysis identified six themes around frontline mental health care providers’ experiences at the primary health care levels during the COVID-19 pandemic: a) interrupted home visits b) lack of government support c) lack of medication d) low clinic attendance e) disruptions in service delivery d) extended medication supply and e) Patient aggression and attacks.

#### Theme 1:Disruptions in service delivery.

Frontline mental healthcare providers reported that the pandemic resulted in significant disruptions to the provision of mental healthcare services. Accordingly, the mental health units at the various outpatient departments were closed with some converted to COVID-19 holding spaces. As a result, mental health care services provision was stopped, reduced, or disrupted in most cases. They talked about experiences of turning away seekers of mental health care services who came to the facilities for review or consultation.

*When the first case was recorded in Ghana, the management of this hospital sent a memo that all the special clinics should be closed, and the staff should be added to the COVID-19 response team. Those in special clinics like psychiatry, ENT, diabetes clinics, sickle cell clinics, etc., said it is not an emergency clinic, so most of their staff were pulled into the COVID-19 response team. Unfortunately, we had to turn away some patients and their caregivers as a result. This affected our clients* ***MH Provider_ 1***

Some noted that, whilst most mental health units were closed or converted into COVID-19 triaging or treatment centers, other PHC facilities withdrew most of the mental health care providers and moved them into other COVID-19 treatment and management centers.

It was revealed that the withdrawal of mental health services was in the initial phase of the pandemic, as most thought this was only for a short while. However, when the pandemic continued and worsened with increasing demand for services, they resumed the provision of mental health care services but at a minimal scope. Consequently, other small spaces were created, and a few staff were assigned to attend to emergency mental health care cases.

*We thought the coronavirus was going to be 2 months something but when we realized that it wasn’t going to go, in August, we went around the hospital and got a single room which we converted into the psychiatric unit. We designated only one staff member to see the patients.* ***MH Provider _ 1***

#### *Theme 2:*Patient aggression and attacks.

As a result of the service disruption through the closure of the mental health units or the scale down in the services provided, it resulted in some patients defaulted, and grew aggressive and violent towards mental health care workers. Some mental health workers recounted that as a result of these disruptions, some patients defaulted and relapsed as well. In this relapsed state, some of the patients presented with aggression and would sometimes even attack them and had to be restrained. This brought some fear to the mental health workers during home or clinic visits because they did not know if their patients were sober or not.

*I can tell you some of the encounters with our clients were unpleasant. From a distant event, before they get close to you, you could sense some bit of unfriendliness and uneasiness in them. In some cases, we had to restrain or sedate some before we got closer and attended to them. Some were off and could not be controlled and with violent and attacking posturing towards us.* ***MH Provider _3***

#### Theme 3:Limited medication and medical supplies for mental health disorders.

Some participants shared that there was already an inadequate supply of medications for mental health patients in the hospital before the pandemic and so the pandemic came to exacerbate the situation. The hospital relied on donor support most of the time to meet the demand for medications as the supply from the health service stores was erratic and inadequate. So when patients could not get medications from the hospital, they were given prescriptions to purchase the medications from pharmacies outside but the challenge was the lack of follow-up to ensure they indeed bought the medications and adhered to the treatment guidelines.

*COVID-19 did not affect the availability of medication; there was already a shortage of medicines, and we’ve had problems with medications for a very long time. COVID-19 only came to make the issue worse as resources were diverted to prevent COVID-19. Here in our facility, we are lucky to be supported by an NGO called Basic Need.* ***MH Provider _7***

#### Theme 4:Low clinic attendance.

The pandemic led to low attendance at the mental health clinics. Several clients defaulted on their review visits because there was a restriction on movement and most of them feared getting infected with the COVID-19 virus at the hospital. Others were afraid of being quarantined because they thought they had COVID-19.

*A major challenge we faced was the low attendance of our clients for review and refill of their medications. As a consequence, we later saw presentations with poor treatment outcomes and some relapses. So attendance during COVID-19 was reduced drastically.* ***MH Provider _3***

Since these patients defaulted on their review visits and medications, many of them returned to a relapsed state.

*Yeah, so because of the restrictions in the country and the lockdown, some of them couldn’t come back for the medications, and eventually some reported in a relapsed state.* ***MH Provider _9***

The low clinic attendance was also explained by the decisions of healthcare workers. It became common practice for patients to be given longer intervals between review visits. Therefore, the number of patients at each visit reduced drastically.

#### Theme 5:Interruption of home visits.

The health workers mentioned that home visits are part of their routine work as a way of checking up on their mental health patients, assessing the home setting of the patient and reducing the defaulter rates. However, the fear of getting infected with the COVID-19 virus, compounded by the lack of adequate funding for home visits, disrupted this essential support service. Some mental health workers mentioned that they reduced the frequency of the home visits and others said they stopped it completely.

*yes, we used to do home visits. However, when Covid came we were all scared of getting infected, so we stopped going for home visits. We stopped. We stopped completely. We were not going for home visits.* ***MH Provider _4***

*so ideally, we used to go on home visits every week, but now sometimes in a month, we go twice but at first, we used to go every week.* ***MH Provider _1***

Some of the mental health workers also mentioned that they did not have adequate financial support to conduct these home visits therefore they visited only nearby homes and even with this, the frequency was reduced.

*Yes, at a point we had to reduce home visits to just the nearby places or visit patients who live close to staff because we did not have allowances for transportation and that was not as frequent as it used to be.* ***MH Provider _4***

#### Theme 6:Extended medication supply.

Most of the mental health workers mentioned that one of the strategies they adopted to reduce the number of hospital visits by patients was to increase the intervals for review visits and medication refills. The usual supply of medications which was sometimes monthly, was extended to about 3 months and patients were required to visit the hospital only when there was an urgent need.

*when they come sometimes, we give them medications that could last them about three months and then when they go, we communicate on the phone to know if the person is okay.* ***MH Provider _8***

### Caregivers of mental health patients

Four themes emerged that characterizes the experiences of caregivers of people living with mental health conditions namely a) denial and withdrawal of facility-based care b) medication refill challenges c) disruption in outreach and follow-up calls and d) isolation and limited community support

#### Theme 1:Denial and withdrawal of facility-based care.

The experiences of caregivers during the COVID-19 pandemic included their inability to easily access healthcare at health facilities. They generally expressed that the imposition of movement restrictions by the government, coupled with decisions of the hospitals to close special clinics, including psychiatric units which were converted into COVID-19 holding bays, obstructed their access to the health facilities for consultation and medication refills and hence resulted in consequences like relapses.

*During COVID-19, there was a lockdown and other movement restrictions, so we could not come to the hospital as required. Especially when the situation was high, the hospitals halted the provision of many services, so the psychiatric unit or clinic was also closed so we missed some review dates. We resumed visiting the hospital at a time there was a little ease with the COVID-19 restrictions.* ***CG_2***

*The only problem I have is that the hospital took this place (psychiatric unit) and used it as a place to keep COVID-19 patients. As a result of this, the psychiatric hospital did not work for some 3–4 months. It was later that they started working at the main hospital building.* ***CG_ 3***

Again, some caregivers hinted that they were officially asked not to come to the hospital by health authorities since resources were channeled into managing COVID-19 and the psychiatric unit was closed as a result of the pandemic.

*For us, the hospital told us not to come because they were very busy with COVID-19. At the hospital, we were asked not to come for some months. There was nothing we could do than wait for the hospital to open for us and that was what we did. We came once but we didn’t see any of the nurses and we went back home. During the period that we were not coming to the hospital, one cannot tell how badly it affected my sister. Denying people hospital care for such a long period is very bad.* ***CG_ 5***

#### Theme 2:Medication refill challenges.

Caregivers also lamented about their inability to refill their medication during the zenith of the COVID-19 pandemic. This was occasioned by the shortage of critical medications in the facilities. Another caregiver stated that their inability to access care during the pandemic caused them to run out of medication stocks. Again, the restrictions on movement in the country caused community pharmacies to reduce their service times; hence it was difficult to procure medication that was unavailable at the hospitals.

*….because they closed the clinic, we were out of medicine and couldn’t restock. The medicine we give her is not something you can easily get at the pharmacy; you need to get a prescription from the hospital and because the clinic was closed, we could not get a prescription to buy the medicine. Aside from that, the community pharmacies also altered their operation times, so it was quite difficult to procure his medication even when prescriptions were issued at the hospital.* ***CG_ 5***

The difficulty in refilling depleted medication was that they were going into relapse and requiring much serious care.

*Again, because the medicines were finished during the period when the hospital (psychiatric unit) was closed down, he became aggressive and difficult to deal with.* ***CG_ 3***

#### Theme 3:Disruption in outreach and follow-up calls.

As part of the mental health care delivery services at the primary healthcare level, home visits were integral. However, COVID-19 disrupted this arrangement, causing a stoppage in home services and other outreach services during the pandemic. This disruption in home visits contributed to a shortage of patients’ medication at home since the nurses could not visit to refill their medication.

*You know, before the pandemic, they used to visit, check on us and anytime they visited, they came with some medications in case we needed a refill. But the pandemic has changed everything. This has since stopped and the only opportunity is to find your way to the hospital for consultation and a refill.* ***CG_ 2***

Participants also noted that the pandemic not only disrupted home visits and other outreach activities but also affected follow-up calls and routine monitoring which the community mental health nurses used to undertake.

*…a call from the nurses was enough to keep you going and know that we were not forgotten but COVID-19 came and changed everything. I don’t remember the last time we got a call from the nurses checking on us. At least, that used to make a difference in our daily struggle, receiving that one call asking how my brother was doing and whether we needed any support or an appointment to visit the hospital. Even calls they hardly pick up calls or respond to us immediately.* ***CG_3***

#### Theme 4: Isolation and limited social support.

Another point identified from the analysis is the experience of isolation and lack of social support. It emerged strongly that caregivers increasingly felt isolated and without support. These experiences were shared to describe the extent to which the pandemic affected support previously received from family and community members. In some accounts, financial support from neighbors and family members has since stopped and telephone calls to check on them and offer emotional support were no longer forthcoming.

*Increasingly, we feel alienated and isolated by our neighbors and other family members. Due to the pandemic, there are so many hardships and so the little financial, emotional, and social support I used to enjoy from neighbors, and calls from extended family members to check on my son are gone. Because I quit my job to care for my son, some community members used to visit and support me financially to buy food and medication, but this has since reduced and I understand because all these people are also struggling. After all, their daily sales in the markets have dipped and I can feel that everyone is trying to survive and fend for their families*. ***CG_1***

Such support, especially the financial support, encouraged them to continue purchasing medications and adhering to treatments. They also noted that some of the social support is used to encourage them to continue with the rehabilitation treatments, especially when they feel exhausted and isolated. With the advent of the pandemic, such support had dwindled and they felt more isolated, with no or little financial support to refill medications.

*Oh!.... hmmm, There were times when you felt you were alone in this situation, you felt like giving up and stopping all medical consultations or unwilling to go for a refill and from nowhere a call came through or a neighbor visited to check on us and ask was the last review session went or ask when next are you going to see the doctor. Some will come and give funds to say that this is for transport or medicines for your next hospital visit. However, all these have ended with the pandemic, so we are alone with little social support now. I don’t blame anyone. I blame this thing called COVID.* **CG_2**

## Discussion

This study sought to describe the experiences of healthcare workers (HCWs) in providing mental healthcare services as well as CG experiences in accessing mental health (MH) services during the COVID-19 pandemic. The study identified six themes that describe the experiences of frontline mental healthcare providers experiences of providing care during the COVID-19 pandemic. On the other hand, four major themes that characterize how caregivers of people living with mental health conditions experienced access and support to care during the pandemic were found.

Evidence shows that the provision of mental health services in Ghana is suboptimal [17], and this situation has deteriorated further during the COVID-19 pandemic [34]. We found that the pandemic exacerbated existing challenges with mental health services in Ghana, leading to disruptions in provision and access to care. Our findings of disruptions in provision and access to care are consistent with the survey conducted by the World Health Organization across 130 countries on the impact of COVID-19 on access to mental health services [26]. The survey showed that the pandemic disrupted critical mental health services in 93% of countries globally, with increasing demand for mental health services. Unavailability of mental health services leads to significant distress and death, a situation that was exacerbated during the pandemic [35]. Thus, the pandemic not only intensified the conditions of those with pre-existing mental health disorders due to a lack of access to care, but it also triggered new mental health disorders [26]. For instance, a significant proportion of African adults experienced anxiety, depression, and insomnia during the pandemic and could not access care [9].

The COVID-19 pandemic highlights the urgent need for comprehensive mental health policies and interventions in Ghana that strengthen telehealth for mental health services. Given that many traditional in-person therapy and counseling options were limited or unavailable due to social distancing requirements and lockdowns, the need for digital and telehealth solutions to access mental health services post-COVID-19 cannot be overemphasized. Evidence suggests that one potential solution to improving access to mental health medications during a pandemic is the expansion of telemedicine and digital health services [36]. Expanding telemedicine services can allow patients to have virtual consultations with healthcare providers, including psychiatrists, from the comfort and safety of their own homes [37]. This service eliminates the need for in-person visits to clinics or hospitals, reducing the risk of exposure to the infection. In addition, patients can easily access medication reviews, therapy management, and health education remotely with the help of telemedicine [38,39]. Further, integrating telemedicine into the healthcare system can maximize the efficiency of healthcare delivery while promoting social distancing measures [40]. This approach can help manage prolonged waiting times, reduce the risk of disease progression, and protect health professionals from infection [40]. Expanding telemedicine services can also benefit individuals in remote geographical areas with limited access to mental health providers [38,39]. Additionally, telemedicine services can address the shortage of mental health specialists by allowing patients to receive care from providers outside of their immediate area [41].

The potential of digital mental health solutions to assuage existing weaknesses in the health system and to address the increased demand for mental health services during the pandemic shows the importance of ensuring equitable and effective care through these digital means [42]. Research has indicated that digital interventions can be effective in the real world, yielding clinical outcomes that are comparable to those of traditional in-person care [43]. Notably, Prescott et al showed that the cost of care was found to be lowest among those utilizing telecoaching, suggesting the potential for these solutions to improve access and affordability [43]. Thus, to increase access to mental health care and to be ready for future pandemics, the government of Ghana, in partnership with private organizations, may invest in digital and telehealth services. However, those digital services need to be tailored carefully to types of mental illness, levels of severity, and the peculiar socio-economic circumstances of potential patients who may use the services.

Furthermore, we found that the strain on resources and the need to prioritize COVID-19 care led to significant disruptions in the supply and distribution of essential medical supplies and medications for treating mental health disorders in Ghana. Our finding is consistent with the scoping review conducted by Kendzerska et al [44]. The review showed that medication supply fluctuated during the pandemic due to the global disruption of supply and transportation chains, manufacturing restrictions due to insufficient raw materials or social distancing measures for workers, stockpiling and panic-buying behaviour in some patients, and higher demands to treat or develop clinical trials for COVID-19 patients. These factors ultimately led to a shortage of medications for patients with chronic conditions, including mental health disorders [44,45]. This disruption in medication access not only exacerbated symptoms but also led to increased anxiety and stress, further compounding the mental health challenges experienced by people with mental health disorders during the pandemic.

To mitigate this challenge, it is important to improve the resilience and flexibility of the supply chain, ensuring that it can adapt to sudden changes in demand. Strategies to achieve this include expanding the supplier base to have a more reliable and higher-quality supply of generic medications, increasing inventory levels, and implementing more robust forecasting and planning systems [46]. Governments and healthcare organizations also need to work closely with manufacturers and distributors to prioritize the production and distribution of critical supplies, including those needed for mental health treatment [47].

## Strengths and limitations

This study presents some notable strengths and limitations worth mentioning. In Ghana and most parts of LMICs, most of the studies to date about mental health and the COVID-19 pandemic have focused on the impact of the pandemic on the mental health of healthcare providers. Thus, this study addresses this critical knowledge gap, and to our knowledge, is about the earliest to shed light on the experiences of accessing mental health care from the perspectives of caregivers and providing care as experienced by frontline mental healthcare workers. The study followed standardized reporting guidelines for conducting and reporting qualitative studies, thus enhancing the rigour of the results. The findings from this study should be interpreted with caution for two reasons. First, although we sought to report on the experiences of mental health conditions through their proxies (caregivers), there is a potential for misrepresentation of the actual experiences of accessing care during the pandemic among people living with mental health conditions. The study did not undertake an analysis based on caregivers of different mental health conditions.

## Conclusion

In this study, we have shared findings on how frontline mental healthcare providers and caregivers of people living with mental health disorders experience some challenges in accessing and providing care respectively. The findings have highlighted some of the existing gaps in the capacity of the health system to provide mental health care, especially in times of a public health outbreak. The findings present opportunities for repurposing health systems to ensure more appropriateness for the delivery of mental healthcare during public health emergencies. Efforts to strengthen the health systems through new programmes and review existing policies or new ones to address the above challenges should be guided by the above findings. Overall, such reform actions will help alleviate the suffering and burden experienced by frontline mental health care workers and caregivers.

## Supporting information

S1 File Check list. (DOCX)
