# Supplementary material for: COVID-19 pandemic and access to mental healthcare: A qualitative study of the experiences of mental healthcare providers and caregivers in Ghana
Source: PLOS Ment Health. 2025 Jul 29;2(7):e0000386. doi: 10.1371/journal.pmen.0000386 (PMC12526265; doi:10.1371/journal.pmen.0000386)
Supplement: S1 File — (DOCX) [file pmen.0000386.s001.docx]

Inclusivity in global research

PLOS’ policy on inclusivity in global research aims to improve transparency in the reporting of research performed outside of researchers’ own country or community and ensures that PLOS publications reporting global research adhere to high standards for research ethics and authorship. Authors of relevant research articles may be asked to complete the questionnaire below, which outlines ethical, cultural, and scientific considerations specific to inclusivity in global research. This questionnaire may be requested when researchers have travelled to a different country to conduct research, if research uses samples collected in another country, research with Indigenous populations or their lands, or if research is on cultural artefacts. Researchers travelling to another country solely to use laboratory equipment will not normally be required to complete the questionnaire. However, the questionnaire can be requested at the journal’s discretion for any submission – if you have been requested to complete this questionnaire by the PLOS journal you submitted to, please do so.

Please complete the questionnaire below and include this as a Supporting Information file with your manuscript. Note that if your paper is accepted for publication, this checklist will be published with your article in the supporting information files. Please ensure that you reference the checklist in the main body of your manuscript. We suggest adding a subsection ‘Inclusivity in global research’ to your Methods section and adding the following sentence: “Additional information regarding the ethical, cultural, and scientific considerations specific to inclusivity in global research is included in the Supporting Information (SX Checklist)”

The questions have been designed to be applicable to a wide range of study types, and there are subsections for both human subjects research and non-human subjects research. If any of the questions are not relevant to your research please mark them as “N/A” as appropriate.

**Ethical considerations, permits and authorship**

*This section is applicable to all research types.*

Provide details as to who granted permissions and/or consent for the study to take place in the Methods section of your manuscript. This should include the names of **all** ethics boards, governmental organizations, community leaders or other bodies that provided approval for the study. If individuals provided approval refer to these people by their role or title but do not list their name(s).

Reported on page number: 8

If there were any deviations from the study protocol after approval was obtained please provide details of these changes in the Methods section of your manuscript.
Did this study involve local collaborators that are residents of the country where the research was conducted or members of the community studied? If you do not have any authors from said communities, please provide an explanation for this below.

Reported on page number: There were no protocol deviations

Yes. Five out of the ten authors are researchers (collaborators) resident in the country of the study, namely Leonard Baatiema(Research lead), Sheba M. P. Kunfah, Kwaku Darko Owusu-Ansa, Ama de-Graft Aikins and Kwadwo A. Koram

**Human subjects research (e.g. health research, medical research, cross-cultural psychology)**

Did you obtain written informed consent from a representative of the local community or region before the research took place? How did you establish who speaks for the community? Details of written informed consent obtained from study participants should be reported separately in the Methods section of your manuscript.

Informed consent was obtained before all participants participated in the study. This study was designed and conducted as part of the career development fellowship for the first author (Dr Leonard Baatiema) who was an LMIC Global Health Fellow under the HBNU Consortium funded by the USA National Institute of Health (NIH). The study received ethical approval from the Ghana Health Service Ethics Review Committee. The study was also supported by the Ghana NCDs Alliance through which the network of people living with NCDs were recruited to participate in this study. The Assembly men and women, local chiefs and traditional leaders were informed about the study and consent and approval were received before the organization of research activities in their communities.

How did members of the local community provide input on the aims of the research investigation, its methodology, and its anticipated outcome(s)?

The Ghana Health Service Ethics Review Committee is represented by different stakeholders from the health sector and beyond and reviewed and made significant inputs to the study protocol and data collection tools and the expected outcomes. The study methodology and anticipated outcomes were also discussed with the National Coordinator of the Ghana NCDs Alliance, a network of patient-based advocates across the various communities in Ghana.

When engaging with the local community, how did you ensure that the informed consent documents and other materials could be understood by local stakeholders?

The consent forms used in this study were based on standardized forms from the Ghana Health Service Ethics Review Committee and these have been used by the local researchers on several previous projects. Trained research assistants were used who were competent in the local languages and thus interpreted the consent forms, participants’ information sheets and the questions to study participants who did not understand the English language.

The study coordinator checks for understanding and clarifies any points that are unclear.

Will the findings of the research be made available in an understandable format to stakeholders in the community where the study was conducted (e.g. via a presentation, summary report, copies of publications, etc.)? Please provide details of how this will be achieved.

The findings from this research will be shared with the Ghana NCDs Alliance and at the annual National Health Summit. We will also summarize the findings in plain, simple language and share them on LinkedIn and X, as well as at local and international conferences. This paper will be published in an open-access journal to widen its chances of attracting huge interest and readership.

**Non-human subjects research using specimens/ animals collected as part of the study, or those housed in archival collections. Examples include archaeology, paleontology, botany and zoology.**

Did the permission you obtained from a local authority to perform the study include an agreement on access to outputs and benefit sharing? This may include procedures to enable fair distribution of the benefits and resources arising from the research performed. Please include any details of Prior Informed Consent and Benefit Sharing Agreements obtained. These may be required by field-specific regulations, for example the Convention on Biological Diversity (CBD) and the associated Nagoya Protocol.

NA

If the material used in your study was imported, please A) provide the year it was imported and B) indicate whether permits were obtained to import/export the materials used, C) provide details of any permits obtained. If this information is not available, please indicate this.

NA

If you used archival specimens, please state how the material used in your study was acquired by the institute it is held in and provide details of any permits obtained for the original excavations/ sample collection. If this information is not available, please indicate this.

NA

How was the potential cultural significance of the materials collected in your study to local communities considered in your research design? Were Indigenous peoples and/or local researchers and institutions involved with archaeological excavations / collection of specimens? If so, please provide a description of their involvement.

NA

If your manuscript includes photographs of human remains please indicate whether authors obtained permission from descendants or affiliated cultural communities to do so.

NA
